# Modeling axial spinal segments of the salamander central pattern generator for locomotion

**DOI:** 10.1186/1471-2202-12-S1-P157

**Published:** 2011-07-18

**Authors:** Andrej Bicanski, Dimitri Ryczko, Jean-Marie Cabelguen, Auke J Ijspeert

**Affiliations:** 1 Biorobotics Laboratory, Ecole Polytechnique Fédérale de Lausanne, Lausanne, Switzerland; 2 Groupe de Recherche sur le Système Nerveux Central, Département de Physiologie, Université de Montréal, Montréal, Québec, H3C 3J7, Canada; 3 INSERM U862 - Neurocentre Magendie, Pathophysiology of Spinal Network Group, Bordeaux Cedex, France

## 

In vertebrates, “central pattern generators” (CPGs) for locomotion are neural networks residing in the spinal cord and brain stem, that transform simple control signals into precisely timed command sequences, e.g. for locomotion, chewing, breathing or digestion. Some studies suggest that the design of the locomotor CPG is evolutionary conservative. Among tetrapods, salamanders resemble the first terrestrial vertebrates and thus represent a key animal from which the evolutionary changes from aquatic to terrestrial locomotion can be inferred. Based on previous work on swimming limbless vertebrates such as the lamprey [1,2], we present a minimal biophysical model of the salamander axial locomotor CPG. Our model is composed of Hodgkin-Huxley model neurons featuring 3 compartments and up to 7 ionic channels. A spinal hemisegment contains 50 sparsely connected excitatory neurons that project to 30 inhibitory neurons. Inhibitory neurons project to all neurons on the contralateral hemisegment, and excitatory neurons further target their contralateral counterparts. Using a single parameter set, this simple segmental network can account for recent electrophysiological and pharmacological data obtained from isolated axial hemisegments and segments of the salamander Pleurodeles waltlii [3]. The model yields oscillating hemisegments with a relation for cycle periods and duty cycles between hemisegments and segments that is similar to the experimental data, as well as correct behavior under the simulated effects of strychnine, apamine, CNQX, riluzole and ZD 2788. The model gives support to the notion that the basic design of the axial locomotor network of the salamander is similar to most axial networks in other vertebrates, albeit with species-specific differences in the cellular mechanisms responsible for segmental bursting. We conclude that this model can serve as a basis for future modeling work on the axial and limb locomotor CPG of the salamander as more salamander-specific data becomes available. This will allow for the study of the modifications in the neural infrastructure necessary for the evolutionary transition from swimming to walking.
